# Effects of Bile Acids and Nisin on the Production of Enterotoxin by* Clostridium perfringens* in a Nutrient-Rich Medium

**DOI:** 10.1155/2018/7276523

**Published:** 2018-02-20

**Authors:** Miseon Park, Fatemeh Rafii

**Affiliations:** Division of Microbiology, National Center for Toxicological Research, U.S. Food and Drug Administration, Jefferson, AR 72079, USA

## Abstract

*Clostridium perfringens *is the second most common cause of bacterial foodborne illness in the United States, with nearly a million cases each year.* C. perfringens* enterotoxin (CPE), produced during sporulation, damages intestinal epithelial cells by pore formation, which results in watery diarrhea. The effects of low concentrations of nisin and bile acids on sporulation and toxin production were investigated in* C. perfringens* SM101, which carries an enterotoxin gene on the chromosome, in a nutrient-rich medium. Bile acids and nisin increased production of enterotoxin in cultures; bile acids had the highest effect. Both compounds stimulated the transcription of enterotoxin and sporulation-related genes and production of spores during the early growth phase. They also delayed spore outgrowth and nisin was more inhibitory. Bile acids and nisin enhanced enterotoxin production in some but not all other* C. perfringens* isolates tested. Low concentrations of bile acids and nisin may act as a stress signal for the initiation of sporulation and the early transcription of sporulation-related genes in some strains of* C. perfringens*, which may result in increased strain-specific production of enterotoxin in those strains. This is the first report showing that nisin and bile acids stimulated the transcription of enterotoxin and sporulation-related genes in a nutrient-rich bacterial culture medium.

## 1. Background

*Clostridium perfringens*, an ubiquitous, anaerobic spore-forming bacterium found in the gastrointestinal tract of humans and animals and in soil, produces various toxins and can cause a variety of mild to severe, even lethal, infections in humans and animals [1]. Some* C. perfringens *type A strains that produce an enterotoxin, CPE, cause food poisoning [2–4]. It is estimated that consumption of food contaminated with enterotoxigenic* C. perfringens *is the second most common cause of bacterial foodborne illness in the United States, with nearly a million cases each year [5]. After food contaminated with the vegetative cells of an enterotoxigenic* C. perfringens *strain is consumed, the bacterium sporulates in the small intestine and produces the enterotoxin CPE, which is released along with the free spores [6]. The enterotoxin binds to the intestinal epithelial cell receptors (claudins) and becomes part of a complex that is oligomerized and inserted into an epithelial cell membrane [7–9]. This results in pore formation, causing cell damage due to increased permeability, manifested by watery diarrhea and stomach cramps [1, 6]. In addition to food poisoning, enterotoxigenic* C. perfringens *type A causes antibiotic-associated diarrhea and non-foodborne sporadic diarrhea [10–13]. The gene encoding the enterotoxin CPE may be located on either the chromosome or the plasmid [14, 15].

CPE enterotoxin is produced only in sporulating cultures but not in vegetative cells [16]. The production of spores is a bacterial survival mechanism [3, 6, 15] that allows* C. perfringens *to survive until environmental conditions change and become favorable for growth. A wide range of environmental and physiological stress signals may trigger sporulation. During the transition from vegetative state to sporulation, the transcription of* spo0A* (gene for sporulation transcription factor), a member of the response regulator control system, and a master regulator, is required [17]. The transcription and translation of a set of RNA polymerase *σ* (sigma) factors that are involved in cell sporulation are also regulated by environmental conditions and are controlled by Spo0A [18]. Genetic analysis has shown that* cpe* transcription is controlled during sporulation of CPE-positive* C. perfringens *strains by three promoters (P1–P3) that are upstream of this gene. Based on consensus promoter recognition sequences, these promoters are dependent on two sporulation-associated RNA polymerase *σ* factors, SigE and SigK [18]. By producing deletion mutants of these two *σ* factors, along with genes for the *σ* factors SigG and SigF, Li et al. and Harry et al. [6, 16] showed that all four *σ* factors are necessary for the production of spores in* C. perfringens *but that only SigE, SigK, and SigF are needed for the production of enterotoxin.

To study spore formation in the laboratory, special sporulation media are often used [19]. However, during infection with food poisoning strains,* C. perfringens* is exposed to nutrient-rich environments in food and in the gastrointestinal tract. In the gastrointestinal tract, they are exposed to bile acids secreted by the liver [20]. In addition,* C. perfringens* strains may come in contact with antimicrobial agents used as preservatives in foods [21]. Bile acids have been reported to have inhibitory or stimulatory effects on the sporulation and production of enterotoxin in various strains of* C. perfringens*, depending on the sporulation medium used for bacterial growth [20, 22, 23]. Nisin, a polycyclic polypeptide of 34 amino acids, has antimicrobial activities against some Gram-positive bacteria, is used in the food industry as a natural preservative, and is allowed in various products [21]. Nisin is reported to delay vegetative cell growth and spore outgrowth of some* C. perfringens* strains* in vitro* [21]. In this study, we have investigated the effects of low concentrations of bile acids and nisin on induction of sporulation, enterotoxin production in medium suitable for vegetative growth, and spore outgrowth. 

## 2. Materials and Methods

### 2.1. Growth of Cultures

*Clostridium perfringens* strain SM101, which is derived from a food poisoning strain, NCTC 8798 [18], and carries an enterotoxin gene on the chromosome, was obtained from Dr. Bruce McClane's laboratory. It was grown in brain heart infusion (BHI) broth under anaerobic conditions (85% N_2_, 10% H_2_, and 5% CO_2_). Colonies were grown anaerobically [24] on blood agar plates (tryptic soy agar containing 5% sheep red blood cells) and used to inoculate BHI broth with or without 1 *μ*g/ml (0.3 *μ*M) of nisin or 100 *μ*g/ml (0.24 mM) of bile acids from Sigma-Aldrich, which contained 50% each of cholic and deoxycholic acid sodium salts. The cultures were incubated at 37°C and samples were withdrawn anaerobically at different intervals during 24 h incubation. The harvested cultures were used for spore and total cell counts, RNA extraction for qRT-PCR, and enterotoxin assays. Other strains of* C. perfringens* [24] were grown similarly in BHI, with or without bile acids and nisin, for the enterotoxin assay.

### 2.2. Enumeration of Total Bacteria and Spores

The bacterial numbers were estimated by preparing serial dilutions of samples taken at intervals, plating on BHI agar, and counting colonies. For the enumeration of spores, 1-ml samples, taken at various intervals, were heated for 20 min at 75°C in a multiblock heating bath (Lab-Line, San Diego, CA) to kill vegetative cells and induce the germination of mature spores. The heat-treated cultures were then serially diluted in sterile 1% peptone, plated on BHI agar, and incubated anaerobically at 37°C. The colonies, representing germinated spores, were counted. Spore and total bacterial counts were performed three times for bacteria grown in BHI in the presence or the absence of nisin and bile acids.

### 2.3. RNA Preparation

*C. perfringens *strain SM101, grown under identical conditions in BHI alone or BHI containing nisin and bile acids, was used to isolate RNA. The RNA was extracted from the cells according to a method previously described [25]. Cells harvested at various intervals after incubation were used for RNA extraction. The cells were centrifuged (15,000 ×g, 10 min, 4°C), washed with 10 mM Tris and 1 mM EDTA (pH 8.0), and suspended in lysozyme buffer [25] containing 10 mg/ml of lysozyme (Sigma). Following incubation for 10 min at room temperature, the cells were centrifuged (15,000 ×g, 10 min, 4°C). The cell pellets were suspended in 50 *μ*l of TE (10 mM Tris, 1 mM EDTA) and mixed with 350 *μ*l of RNAWIZ from Ambion (Grand Island, NY). 75 *μ*l of chloroform was added to the samples, which were incubated for 30 min on ice. The samples were then centrifuged (15,000 ×g, 30 min, 4°C) and the clear phases containing RNA were removed to different tubes. The RNA samples were applied to an RNeasy Mini Kit from Qiagen, Inc. (Valencia, CA), to further purify RNA. Contaminating DNA was removed using a Turbo DNA-free kit (Ambion). A Nanodrop ND-1000 spectrophotometer (NanoDrop Technology, Wilmington, DE) was used to determine the quantity and quality of total RNA. To avoid RNA degradation, the RNA was stored at −80°C and used for qRT-PCR within a week of extraction.

### 2.4. Quantitative Reverse Transcriptase PCR (qRT-PCR)

Primers used for qRT-PCR (Table 1) were prepared based on the published sequences of the chromosomal enterotoxin gene of* C. perfringens* SM101 and other different genes involved in sporulation of SM101 in the GenBank. The RNA extracted from the treated and untreated cultures was used to synthesize cDNA by using Superscript™ III First-Strand Synthesis SuperMix (Invitrogen, Carlsbad, CA). qRT-PCR experiments were performed to compare the effects of the compounds on gene transcription by using SYBR® GreenER™ qPCR SuperMix (Invitrogen). Reaction mixtures were prepared on ice and contained 2x SYBR GreenER qPCR universal mix, 2 *μ*M of each forward and reverse primer, and 1 ng of the cDNA template in 10 *μ*l of reaction mix [25]. The CFX96 real-time PCR detection system (Bio-Rad, Hercules, CA) was programmed for the amplification of the genes, using the following parameters: 50°C for 10 min, 95°C for 8.5 min for the inactivation of uracil DNA glycosylase and activation of DNA polymerase, followed by 40 cycles of 95°C for 15 sec and 58°C for 30 sec to amplify cDNA. To detect nonspecific amplification, melting curves were monitored at 65–95°C (1°C per 5 sec). A 16 S rRNA gene of equivalent size was amplified as a reference RNA for normalization using 1 ng of cDNA. To rule out genomic DNA contamination, reaction mixtures were prepared without reverse transcriptase. To insure lack of contamination of reagents and tubes by nucleic acids, reaction mixtures without templates were included as additional controls. All the PCR reactions were run in triplicate for RNA extracted from three different cultures of each of the samples taken at various times. The cycle thresholds (CT) for 16S RNA and each of the amplified genes were determined. The relative level of expression of each of the genes with respect to 16 S RNA was calculated by the 2^−ΔCT^ method. The relative level of expression of each type of RNA from BHI cultures containing bile acids and nisin, compared to RNA from samples grown with BHI alone, was calculated by the 2^−ΔΔCT^ method, according to the Real-Time PCR Application Guide from Bio-Rad, to determine the effect of the additive on expression of each gene [25].

### 2.5. Assays for Enterotoxin

For detection of the effects of different compounds on the production of enterotoxin, the* C. perfringens *enterotoxin ELISA kit from Creative Diagnostics (Shirley, NY) and* C. perfringens* enterotoxin test from TechLab (Blacksburg, VA), which contain monoclonal and polyclonal antibodies, respectively, in the kit, were used. The monoclonal and polyclonal antibodies against epitopes of the* C. perfringens *enterotoxin are bound to the surfaces of the wells provided in the kits. The* C. perfringens *samples harvested at different intervals were centrifuged (15,000 rpm for 10 min) and the supernatant from each culture was diluted 1 to 5 and added to the wells. The supernatant of* C. perfringens* ATCC 13124 was used as a negative control, and a special reagent interacting with the CPE antibody provided in the ELISA kits was used as a positive control. Following the addition of biotinylated anti-enterotoxin antibody from the kit to the wells, they were incubated at room temperature. After 1 h of incubation, the contents were washed to remove unbound materials. Streptavidin-peroxidase conjugate from the kit was added to the wells and incubated at room temperature for 30 min. The unbound streptavidin-peroxidase conjugates were removed and wells were washed before the addition of the substrate, a solution of hydrogen peroxide/tetramethylbenzidine (TMB). The intensity of blue color development was measured with a spectrophotometer at 620 nm and used to indicate the presence of enterotoxin.

### 2.6. Effects of Bile Acids and Nisin on Spore Outgrowth and Vegetative Growth by Spectrophotometric Analysis

Bacterial spores were prepared from 300 ml of culture according to the method of Novak et al. [3] and stored in 1% peptone or water. The culture was centrifuged at 5000 ×g for 10 min, treated with 10 ml of a solution containing 200 *μ*g/ml lysozyme and 200 *μ*g/ml of trypsin, and incubated for 4 h at 37°C with horizontal shaking. SDS (final concentration 1%) was added to the culture, which was returned to the shaker. The culture was centrifuged and the pellet was suspended in 10 ml of water and left at room temperature overnight. The cells were centrifuged and the pellet was resuspended in 1 ml of water. The purity of the spores was verified with a phase-contrast microscope. The spores were suspended to a final concentration of 10^5^ spores/ml BHI medium, with or without 100 *μ*g/ml of bile acids, and 1 *μ*g/ml of nisin, in triplicate. The spores were then heat-treated for 20 min at 75°C and dispensed in the wells of 48-well plates in BHI medium, with or without 100 *μ*g/ml of bile acids and 1 *μ*g/ml of nisin, in triplicate. Similar experiments were performed for spectrophotometric analysis of vegetative growth using a diluted (10^−3^) overnight culture of cells as inoculum instead of spores. The cultures were placed under anaerobic conditions, overlaid with mineral oil, and inserted into the spectrophotometer for detection of the kinetics of growth. Statistical analysis for different experiments was performed using Excel.

## 3. Results

### 3.1. Effects of Bile Acids and Nisin on the Production of Enterotoxin in* C. perfringens* SM101

**Figure 1:**
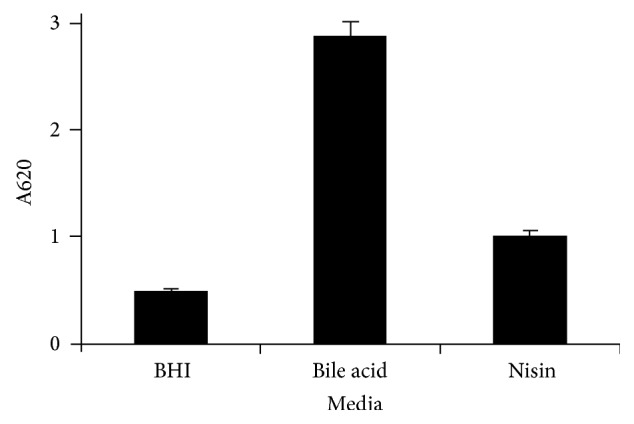
Production of enterotoxin in* C. perfringens* SM101 in BHI, with or without low concentrations of bile acids and nisin. The data represent the average of three independent experiments.

The enterotoxin produced in cultures of* C. perfringens* SM101 in BHI alone or in BHI containing bile acids and nisin was measured three times by ELISA. After 24 h of incubation, the highest amounts of enterotoxin found in all three experiments were observed in cultures containing bile acids, followed by the amounts produced in cultures containing nisin (Figure 1, Supplementary Figure 1A).

### 3.2. Comparison of Growth and Spore Formation in Differently Treated Cultures

**Figure 2:**
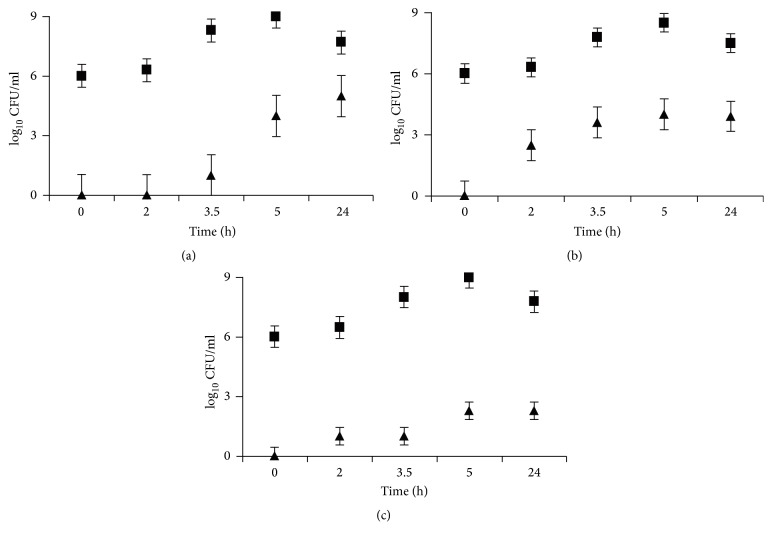
log_10_ of total number of vegetative cells and spores (■) and spores alone (▲) of* C. perfringens* SM101 produced in BHI medium without (a) and with bile acids (b) and nisin (c). The data represent the averages of three experiments.

To determine the effects of bile acids and nisin on growth, spore production, and germination in* C. perfringens* SM101, the cells and germinating spores in samples taken at various intervals during 24 h incubation, in BHI alone or BHI containing either 100 *μ*g/ml (0.24 mM) of bile acids or 1 *μ*g/ml (0.3 *μ*M) of nisin, were counted and compared.  Figure 2 shows the log of the total number of colonies produced from the samples and the germinating spores that were plated at different times from the cultures grown in BHI with or without bile acids and nisin. There has been a report of heat-sensitive or nongerminating spores, so the spore counts reflect only the germinating spores that produced visible colonies [20].

The total number of* C. perfringens* cells increased equally with time in all three cultures during logarithmic growth. Bile acids and nisin, however, delayed the initial phase of growth. This delay was determined by observing the kinetics of growth spectrophotometrically, in which the OD of the culture was measured continuously (Figure 2S). The number of spores also increased. In all three experiments, the ratio of germinating spores/total cell numbers was higher in the early stage of growth in cultures containing bile acids and nisin than in the cultures containing BHI alone.

### 3.3. Comparison of Transcription of Sporulation-Related Genes

**Figure 3:**
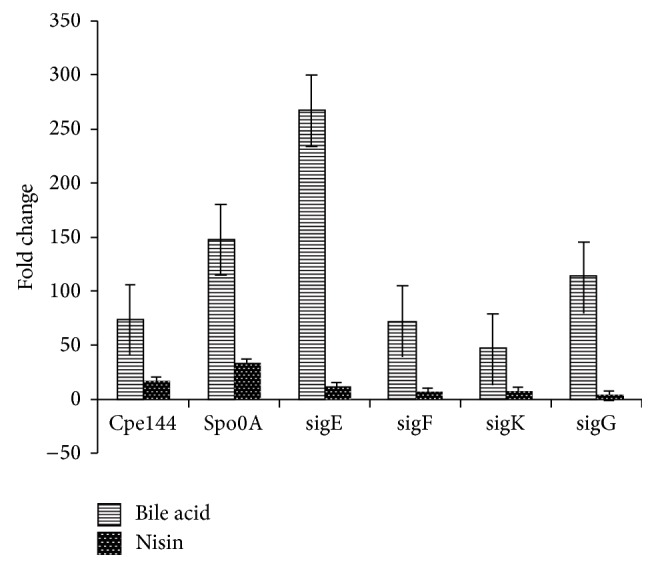
Representative results of the fold change in the transcription of sporulation-related genes from* C. perfringens *SM101 grown in BHI in the presence of bile acids (lined bars) or nisin (dotted bars) after 3.5 h of incubation in comparison with transcription of these genes in bacteria grown in BHI alone. Samples taken at 2 and 5 h also showed upregulation of the same genes.

The expressions of* spo0A*,* sigF*,* sigE*,* sigK*,* sigG*, and the enterotoxin gene* cpe*, which are involved in the production of spores and enterotoxin in* C. perfringens *[6, 17, 18], were measured at intervals during 24 h incubation. The cycle thresholds for each of the genes amplified in cultures grown in BHI alone and those grown with bile acids and nisin were determined. The transcription level of each gene in each of the cultures incubated for various times was normalized with respect to 16S RNA and the fold increase in transcription of each gene with respect to 16S RNA [25] was determined by calculating 2^−ΔCT^. The level of transcription of each gene at a specific time in cultures containing bile acids and nisin was compared with the level of the transcription of the same gene in BHI alone at that time by determining 2^−ΔΔCT^ to show the effect of additives (i.e., bile salts and nisin) on transcription.  Figure 3 shows the fold changes in transcription levels of various genes in cultures of* C. perfringens *SM101 grown for 3.5 h in BHI containing additives in comparison with cultures grown in BHI alone. In general, in early growth phase samples taken at 2, 3.5, and 5 h, the expression of the sporulation-related genes and* cpe* increased in the presence of the additives. Although the increase in expression varied in different experiments, the addition of bile acids and nisin consistently resulted in enhanced expression of* cpe*,* spo0A*,* sigF, sigE*,* sigG*, and* sigK *genes.

### 3.4. Effects of Bile Acids and Nisin on Spore Outgrowth of* C. perfringens* SM101

**Figure 4:**
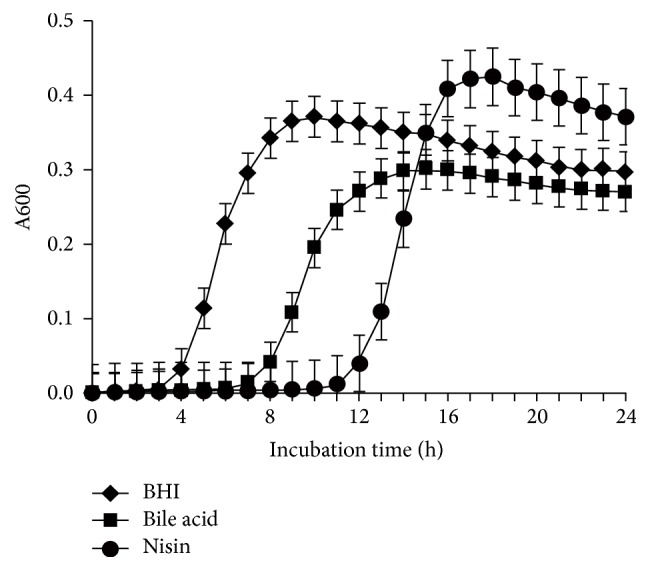
Effect of low concentrations of bile acids and nisin on* C. perfringens* SM101 spore outgrowth.

Spore outgrowth was measured by inoculating* C. perfringens* spores into BHI with or without low concentrations of bile acids and nisin.  Figure 4 shows the kinetics of outgrowth of spores in each of the cultures. Bile acids and nisin delayed the outgrowth of newly germinated spores. In BHI cultures without additives, the increase in growth was observed earlier than in the other two cultures, followed by the culture containing bile acids. The greatest delay in growth after spore germination occurred in the cultures containing nisin.

### 3.5. Effects of Bile Acids and Nisin on Production of Enterotoxin in Other Isolates of* C. perfringens*

The effects of bile acids and nisin on enterotoxin production in enterotoxigenic* C. perfringens* isolates were also examined, in both Duncan and Strong medium and BHI. Out of several isolates tested, either or both of the additives enhanced enterotoxin production in some strains but inhibited or had no effect on others (Supplementary Figure 3).

## 4. Discussion

Ingestion of food containing enterotoxigenic* C. perfringens* is the cause of the second most common bacterial foodborne illness in the United States. The enterotoxin produced during sporulation damages intestinal epithelial cells, which results in cramps and watery diarrhea. Bile acids previously have been shown to stimulate spore formation and enterotoxin production in some, but not all,* C. perfringens* strains in sporulation medium [20]. We found that bile acids and, to a lesser extent, nisin also stimulated spore formation and enterotoxin production in a rich medium that supported the vegetative growth of this bacterium. We detected that, in BHI medium, low concentrations of bile acids and nisin stimulated early transcription of sporulation-related genes, which may be the reason for enhanced spore formation during the early bacterial life cycle, and bile acids had the greatest effect on transcription, spore formation, and enterotoxin production. Both compounds also delayed spore outgrowth in BHI medium; nisin had the most inhibitory effect.* C. perfringens* enterotoxin has been detected in sporulation medium 2 h after inoculation in variable amounts [26]. Variation in sporulation frequency in different cultures has been reported previously [26] and was also observed in our experiments using enriched (BHI) medium. However, we found that bile acids and nisin had direct effects on sporulation, toxin production, and spore outgrowth in* C. perfringens* SM101.

To determine the time when sporulation development was affected by added chemicals, samples taken at intervals during 24 h incubation were analyzed. The increase in the early sporulation rate and enhancement of enterotoxin production were the results of stimulation of the transcription of enterotoxin and sporulation-related genes by the additives as early as 2 h after incubation. In* C. perfringens, spo0A* gene transcription is required for endospore formation and enterotoxin production [17, 27]. The transcription of this gene was stimulated in the presence of bile acids and nisin, as was evident by the fold increase in transcription in cultures containing the additives in comparison with the cultures in BHI alone. Similarly, the transcription of* cpe*, other sporulation-specific sigma factor genes* sigE* and* sigK,* and the alternative sigma factor genes* sigF *and* sigG* [15, 16] also was enhanced in comparison with cultures without these chemicals. All four sigma factors, SigE, SigF, Sig G, and Sig K, control sporulation in* C. perfringens* [15, 16]. The transcription of* cpe* is dependent on SigE and SigK; and three promoters upstream of* cpe* are similar to the consensus SigE and SigK-dependent promoters [6]. Because SigF controls* sigK* and* sigE *expression, transcription of* cpe* also depends on SigF [6]. Yasugi et al. [28], using microarray analysis, showed that all four sigma factors were upregulated in the presence of sodium deoxycholate in* C. perfringens* grown with Caco-2 cells in Dulbecco's modified Eagle's medium (DMEM) after 4 h of incubation. The reason for the enhanced transcription of sporulation-related genes by bile acids and nisin found in* C. perfringens* grown in BHI in this study is not known. Yasugi et al. [28] found that deoxycholate enhanced phosphorylation of the Spo0A protein in* C. perfringens,* which was cocultured in intestinal epithelial Caco cells, and concluded that deoxycholate may have facilitated phosphorylation of Spo0A and activation of Spo0A-controlled genes. Whether the presence of bile acids and nisin in BHI also enhances the phosphorylation of the Spo0A protein that controls sporulation-related genes [6] merits investigation.

Since growth conditions affect sporulation of different strains of* C. perfringens*, conflicting reports in the literature for the effects of bile acids on spore formation may be related to differences in the media and strains used. Hickey and Johnson [23], growing* C. perfringens *in Duncan and Strong [19] medium containing 0.3 to 6.5 mM sodium cholate or sodium deoxycholate, found that direct spore counts declined only in concentrations of 3.5–6.5 mM sodium cholate and deoxycholate, but found that concentrations of >1.2 mM deoxycholate reduced the count of heat-resistant spores. However, Heredia et al. [20], using the Defined medium (D medium) of Sacks and Thompson [29], reported that increasing the concentration of sodium cholate (0.125–2 mg/ml) had a stimulatory effect on enterotoxin and spore production in some strains of* C. perfringens* but failed to stimulate the production of heat-resistant spores in other strains. A concentration of 1.8 mM sodium deoxycholate reduced the number of heat-resistant spores [23]. Because the production of enterotoxin begins in early stages of sporulation (Stage III), some bile salts could induce sporulation but prevent the development of mature spores [20].

de Jong et al. [22] reported inhibition of sporulation of most* C. perfringens* strains in Duncan and Strong (DS) medium containing 0.05% bile acids. Guerlava et al. [30] used concentrations of 0.15–3 *μ*M (50–1000 IU/ml) of nisin and found that 0.15 *μ*M lengthened the lag phase but did not inhibit growth. Concentrations of 0.6 *μ*M nisin and above had dose-dependent inhibitory effects [21, 30]. Udompijitkul et al. [21] showed that 1 *μ*M nisin had an inhibitory effect on vegetative cells and arrested the growth of vegetative cells for 6 hours, but growth then resumed. Five *μ*M was inhibitory over 24 hours in their medium. We found that low concentrations of bile acids and nisin had strain-specific effects on the production of enterotoxin by different* C. perfringens* isolates grown in either BHI or DS medium. Variation was also observed in enterotoxin production in different strains assayed at various times. Addition of either or both of these compounds increased enterotoxin production in some other strains tested. The enterotoxin production in other strains was not affected or decreased (Supplementary Figure 3). Heterogeneity of the* C. perfringens* enterotoxins and its production from different strains have been reported previously [20, 31], which may be related to the genomic variations observed during study of a large number of* C. perfringens* strains [32, 33].

The concentrations of bile acids (0.24 mM) and nisin (0.3 *μ*M) used in our experiment did not inhibit spore production in BHI media; rather, they stimulated the transcription of sporulation-related genes and spore formation in rich (BHI) medium. The number of germinating spores detected in the early growth phase was also higher, although it may not be reflective of all spores produced in the early stage, since heat-sensitive, nongerminating spores have been reported in other studies [20].

The total bacterial counts after 24 h of incubation were similar in cultures with and without additives, indicating that the concentrations of additives used were not inhibitory. Similar to the observation of Liu et al. [34],* C. perfringens* SM101 had a long lag phase even while growing in BHI without bile acids and nisin (Figure 2 and Supplementary Figure 2S). The lag phase was longer than that observed for* C. perfringens* grown in fluid thioglycolate medium (FTH), which contains sodium thioglycolate and L-cysteine [21]. Differences in the concentrations and range of metabolites also have been reported between* C. perfringens* grown in BHI and thioglycolate medium [35]. It is not clear if the difference in the length of the lag phase in the two media is related to the more reduced state of the FTH medium or to other factors that affect the preparation of* C. perfringens *to transition from stationary to exponential phase in these two media. Transitory transcription of numerous genes during lag phase has been shown in other bacteria [36].

In our experiments, both bile acids and nisin delayed growth of* C. perfringens *SM101 at the early stage of incubation, as evidenced by the increase in the lag phase in the kinetics of growth; nisin had a greater effect (Supplementary Figure 2S). An increase in the lag phase of* C. perfringens* grown with nisin also has been shown previously [21, 30]. Udompijitkul et al. [21] showed growth inhibition and a long lag phase for* C. perfringens* SM101 grown in FTH medium containing 1 *μ*m nisin. They also showed that 0.1 *μ*M of nisin completely inhibited the spore outgrowth of* C. perfringens* SM101 in TGY (3% trypticase, 2% glucose, 1% yeast extract, and 0.1% L-cysteine) medium, but high concentrations of nisin in cooked meat did not inhibit the growth of* C. perfringens* spores, which the author attributed to the loss of antimicrobial activity of nisin in meat [21].

We found that a concentration of 0.3 *μ*M nisin delayed but did not prevent the outgrowth of spores in BHI medium (Figure 4). Although various factors, including absorption, degradation, and food intake, affect the concentrations in the GI tract of bile acids [37] and nisin [38], the concentrations used in this study of bile acids (0.24 mM) and nisin (0.3 *μ*M) were below the inhibitory concentrations for some* C. perfringens* strains tested [21, 23, 30, 39]. The bile acid concentration used was within the range found in the GI tract of humans [37]. It is conceivable that a concentration of 0.3 *μ*M (1 *μ*g/g of GI content) could be achieved through the ingestion of a food containing nisin. The concentration of nisin in the GI tract of human flora-associated rats is correlated with the nisin intake [38]. 0.3 *μ*g/ml of nisin, however, inhibited the growth of some other* C. perfringens* field isolates in BHI (data not shown).

## 5. Conclusions

A wide range of environmental and physiological signals may initiate sporulation, which accompanies enterotoxin production in* C. perfringens *[3, 6, 15]. Our data show higher enterotoxin production in a rich medium resulting from the enhanced transcription of sporulation-related genes by low concentrations of nisin and bile acids in some strains. However, the implication for pathogenesis when small amounts of this food preservative (nisin) are ingested with food, or when the strains are exposed to bile acids in the gastrointestinal tract, is not known at this time. Conditions of the growth medium affect sporulation and enterotoxin production [40, 41], so it is not clear if the exposure to secreted bile acids and residual nisin by the consumption of foods containing preservatives has any effect on* in vivo* stimulation of enterotoxin, as is found* in vitro* in some strains. Enhancement of sporulation by these sporulation-promoting compounds was highly strain-dependent, which may be related to the wide genetic variation found among different* C. perfringens* strains [32, 33]. To our knowledge, this is the first report that shows that nisin and bile acids may enhance transcription of genes involved in sporulation and enterotoxin production in some* C. perfringens* strains in nutrient-rich bacterial culture medium.

## Figures and Tables

**Table 1:** Forward (for) and reverse (rev) primers used for the PCR and qRT-PCR amplification of enterotoxin genes and sigma factors in *C. perfringens.*

Primer	Sequence, 5′ to 3′	Size, base pairs
cpe for	TCCAATGGTGTTCGAAAATG	144
cpe rev	GGTTCCCCTAATATCCAACCA	
SigK for	TGGAGATGTTGAGGCAAAAA	195
SigK rev	GCTGCATATGTTGCAAGTCG	
SigE for	GCTTGCAACCTATGCATCAA	128
SigE rev	AAAGTTCATTTCCATCCCAATC	
Spo0A for	GCAAAAGATGGATTGGAAGC	173
Spo0A rev	TTGTCTTGTCCAACAGCAGA	
SigF for	GGAACGCCGGTTCTCTTAAT	172
SigF rev	CAAGCATTTTTGCAACTTGA	
SigG for	CTTTATTTGAGCCTATTTATTATG	99
SigG rev	TATTTTCAAGCCAACTATCA	
Vir X for	TGGAAAAGAATTCGTATTCACTGTA	100
Vir X rev	TCTTGCTTTTCTGCAAGCTG	
16S RNA for	TGGGGAGCAAACAGGATTAG	212
16S RNA rev	TAAGGTTCTTCGCGTTGCTT	

## Supplementary Materials

Supplementary Materials Supplementary Figure 1. ELISA results showing the effects of bile acids and nisin on enterotoxin production in* C. perfringens* SM101. The supernatant of* C. perfringens* ATCC 13124 was used as an enterotoxin-negative control. The positive control was provided in the ELISA kit by the manufacturer. – control shows supernatant of strain 13124, and “+ control” shows the reaction of diluted* C. perfringens* enterotoxin in a buffered protein solution provided in the ELISA kit as a positive control. Supplementary Figure 2: spectrophotometric analysis of the effect of low concentrations of bile acids and nisin on the vegetative growth of* C. perfringens* SM101 in BHI medium. Supplementary Figure 3: effects of bile acids and nisin on the production of enterotoxin in different strains of* C. perfringens* grown in BHI medium. Other strains of* C. perfringens* that were tested but did not show any effect are not included. The sources of strains are as follows: NRRL B-23743 (clinical strain, disease unknown), 1407 (canine colitis), 1420 and 1421 (canine enteritis), CP-B17 and CP-B31 (unrelated soil isolates), and CP-B46 (food isolate/chicken). CP-B31 and CP-B46 were not assayed for plasmid isolation. No plasmid was detected in 1420. The rest of the strains contained variously sized plasmids. Plasmid profiles and genetic diversity of these strains have been shown previously [32]. Except for 1420 and 1421 that were located on the same cluster of PFGE dendrograms, the rest of the isolates belonged to diverse clusters on dendrograms. “+” shows the reaction of diluted* C. perfringens* enterotoxin in a buffered protein solution that was provided in the ELISA kit as a positive control.
