# Supplementary material for: Effects of Bile Acids and Nisin on the Production of Enterotoxin by Clostridium perfringens in a Nutrient-Rich Medium
Source: Int J Microbiol. 2018 Feb 20;2018:7276523. doi: 10.1155/2018/7276523 (PMC5838459; doi:10.1155/2018/7276523)

SUPPLEMENTARY FIGURE 1. ELISA results showing the effects of bile acids and nisin on enterotoxin production in *C. perfringens* SM101. The supernatant of *C. perfringens* ATCC 13124 was used as an enterotoxin-negative control. The positive control was provided in the ELISA kit by the manufacturer. – Control, supernatant of strain 13124, "+ control" shows the reaction of diluted *C. perfringens* enterotoxin in a buffered protein solution provided in the ELISA kit as a positive control.

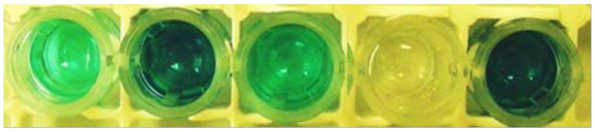

BHI   Bile Acid   Nisin   - control   + control

SUPPLEMENTARY FIGURE 2. Spectrophotometric analysis of the effect of low concentrations of bile acids and nisin on the vegetative growth of *C. perfringens* SM101 in BHI medium.

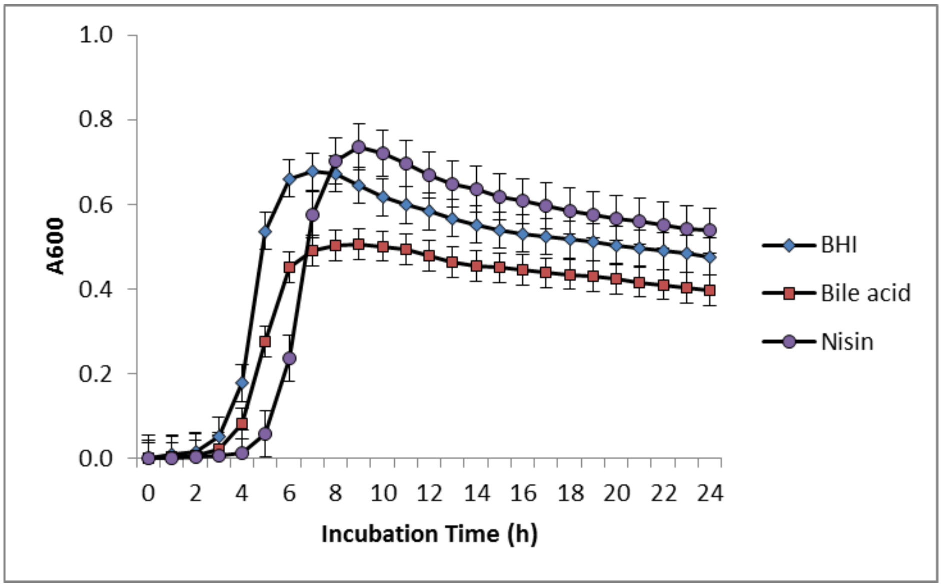

SUPPLEMENTARY FIGURE 3. Effects of bile acids and nisin on the production of enterotoxin in different strains of *C. perfringens* grown in BHI medium. Other strains of *C. perfringens* that were tested but did not show any effect are not included. The sources of strains are as follows: NRRL B-23743 (clinical strain, disease unknown), 1407 (canine colitis), 1420 and 1421 (canine enteritis), CP-B17 and CP-B31 (unrelated soil isolates) and CP-B46 (food isolate/chicken). CP-B31 and CP-B46 were not assayed for plasmid isolation. No plasmid was detected in 1420. The rest of the strains contained variously sized plasmids. Plasmid profiles and genetic diversity of these strains have been shown previously [33]. Except for 1420 and 1421 that were located on the same cluster of PFGE dendrograms, the rest of the isolates belonged to diverse clusters on dendrograms. "+" shows the reaction of diluted *C. perfringens* enterotoxin in a buffered protein solution that was provided in the ELISA kit as a positive control.

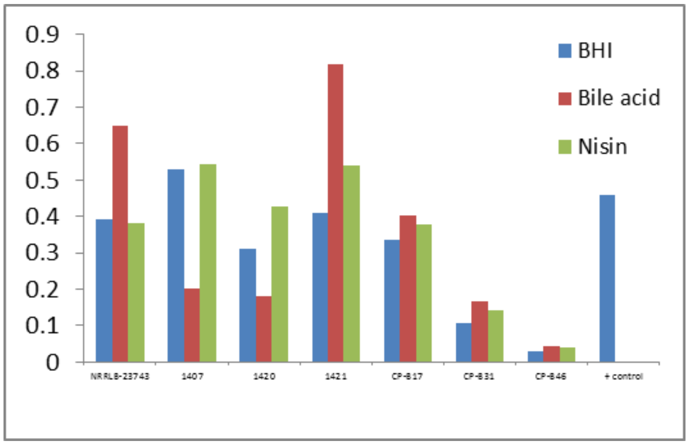

Supplement: Supplementary Materials — Supplementary Figure 1. ELISA results showing the effects of bile acids and nisin on enterotoxin production in C. perfringens SM101. The supernatant of C. perfringens ATCC 13124 was used as an enterotoxin-negative control. The positive control was provided in the ELISA kit by the manufacturer. – control shows supernatant of strain 13124, and “+ control” shows the reaction of diluted C. perfringens enterotoxin in a buffered protein solution provided in the ELISA kit as a positive control. Supplementary Figure 2: spectrophotometric analysis of the effect of low concentrations of bile acids and nisin on the vegetative growth of C. perfringens SM101 in BHI medium. Supplementary Figure 3: effects of bile acids and nisin on the production of enterotoxin in different strains of C. perfringens grown in BHI medium. Other strains of C. perfringens that were tested but did not show any effect are not included. The sources of strains are as follows: NRRL B-23743 (clinical strain, disease unknown), 1407 (canine colitis), 1420 and 1421 (canine enteritis), CP-B17 and CP-B31 (unrelated soil isolates), and CP-B46 (food isolate/chicken). CP-B31 and CP-B46 were not assayed for plasmid isolation. No plasmid was detected in 1420. The rest of the strains contained variously sized plasmids. Plasmid profiles and genetic diversity of these strains have been shown previously [32]. Except for 1420 and 1421 that were located on the same cluster of PFGE dendrograms, the rest of the isolates belonged to diverse clusters on dendrograms. “+” shows the reaction of diluted C. perfringens enterotoxin in a buffered protein solution that was provided in the ELISA kit as a positive control. [file 7276523.f1.pdf]
